# Hindgut Microbiota Reflects Different Digestive Strategies in Dung Beetles (Coleoptera: Scarabaeidae: Scarabaeinae)

**DOI:** 10.1128/AEM.02100-20

**Published:** 2021-02-12

**Authors:** Kathryn M. Ebert, William G. Arnold, Paul R. Ebert, David J. Merritt

**Affiliations:** a School of Biological Sciences, University of Queensland, Brisbane, Australia; University of Illinois at Chicago

**Keywords:** symbiosis, Australian endemic genera, gut morphology, detritivore, *Desulfovibrio*, coevolution

## Abstract

Gut microbes play an important role in the biology and evolution of insects. Australian native dung beetles (Scarabaeinae) present an opportunity to study gut microbiota in an evolutionary context as they come from two distinct phylogenetic lineages and some species in each lineage have secondarily adapted to alternative or broader diets. In this study, we characterized the hindgut bacterial communities found in 21 species of dung beetles across two lineages, using 16S rRNA sequencing. We found that gut microbial diversity was more dependent on host phylogeny and gut morphology than specific dietary preferences or environment. In particular, gut microbial diversity was highest in the endemic, flightless genus *Cephalodesmius*, which feeds on a broad range of composted organic matter. The hindgut of *Cephalodesmius* beetles harbors a highly conserved core set of bacteria, suggesting that the bacteria are symbiotic. Symbiosis is supported by the persistence of the core microbiota across isolated beetle populations and between species in the genus. A coevolutionary relationship is supported by the expansion of the hindgut to form a fermentation chamber and the fermentative nature of the core microbes. In contrast, Australian species of the widespread dung beetle genus *Onthophagus* that specialize on a single food resource, such as dung or fungus, exhibit minimal food processing behavior and have a short, narrow hindgut and a variable gut microbiota with relatively few core bacterial taxa. A conserved, complex gut microbiota is hypothesized to be unnecessary for this highly mobile genus.

**IMPORTANCE** Dung beetles are a very important part of an ecosystem because of their role in the removal and decomposition of vertebrate dung. It has been suspected that symbiotic gut bacteria facilitate this role, a hypothesis that we have explored with high-throughput barcoding. We found that differences in hindgut morphology had the greatest effect on the bacterial community composition. Species with a hindgut fermentation chamber harbored a distinctly different hindgut community compared to those species with a narrow, undifferentiated hindgut. Diet and phylogeny were also associated with differences in gut community. Further understanding of the relationships between dung beetles and their gut microbes will provide insights into the evolution of their behaviors and how gut communities contribute to their fitness.

## Abstract (precis)

Dung beetles are a very important part of an ecosystem because of their role in the removal and decomposition of vertebrate dung. It has been suspected that symbiotic gut bacteria facilitate this role, a hypothesis that we have explored with high-throughput barcoding.

## INTRODUCTION

The insect gut can be colonized by various microorganisms, but the composition, abundance, and stability of microbial taxa vary considerably across the diverse orders of insects (1). The observed differences in gut bacterial communities can be attributed to several factors, including host diet, phylogeny, environment, gut morphology, and behavior (2–6). Microbes are known to be of functional significance, especially in insects with nutritionally limited diets or difficult-to-digest diets (1). Often, insects with specialized diets, such as honeybees (Apis mellifera) have a small number of specialized core gut taxa (4, 7). In contrast, insects with broad diets, such as omnivores and detritivores, have gut communities that are diverse, and some insects can have hundreds of taxa (4, 8–10). Detritivores, in particular, share a distinct and diverse microbial gut community, even though they occur in divergent taxonomic groups, which suggests that a specialized microbiota is required to consume decaying organic material (2).

While many insects have an undifferentiated gut morphology, many of the detritivores have enlarged regions in the hindgut (11–16). Insects such as termites (16), detritus-feeding fly larvae (15), and scarab beetle larvae (11, 17) all have a dilated hindgut region that forms an anoxic fermentation chamber. This provides a suitable microhabitat for anaerobic microbes to establish residence and in turn aid with the digestion of plant polysaccharides and other lignocellulosic matter. In several species of soil-dwelling scarab beetle larvae (Melolonthinae and Cetoniinae), the hindgut microbial community has been found to be highly diverse and many of the gut microbes are consistently present, suggesting a level of symbiosis (11, 17, 18). Most of these studies have focused on the gut communities in the larval stages (19), while comparative studies of adult scarab beetles are limited.

Among the scarabs, the true dung beetles (Scarabaeidae: Scarabaeinae) are an ecologically important group because of their association with vertebrate dung. Dung feeding is a specialized form of detritivory, and it is suspected that dung beetles rely on gut microbiota to aid with digestion as dung is considered to be a nutritionally limited food source (20–23). The dung beetle larvae, in particular, have a hindgut fermentation chamber, as is seen in other subfamilies of scarab beetle larvae (23), yet only a few studies have investigated gut microbiota in dung beetles. Many have focused on transmission of microbes between adults and larvae in a few selected species (24, 25). A comparative study of adult beetles from five different families included four dung beetle species from the genus *Onthophagus* (3). These dung beetles had a more diverse gut microbial community than all other beetle families, but the microbiota were highly variable (3). Another study, also investigating *Onthophagus* spp., found that their gut microbiota shared some core elements yet was significantly influenced by the local environment when the insects were introduced to new locations (5). In the dung beetle genus *Euoniticellus*, adult male and female gut communities were significantly different, and the composition of the female gut was more similar to that of the larval gut (26). This difference may reflect the fact that in this species, only the female is engaged in preparing the brood material. Two African congeneric dung beetle species (*Pachysoma*), both with atypical diets—one a dry-dung feeder and the other a plant detritus feeder—had a small core microbiota; however, the gut bacterial compositions differed between the two species. The detritivorous species had the greater bacterial diversity overall (27).

Australian native dung beetles (Scarabaeinae) are ideal for a comparative study of gut microbiota in an evolutionary context. From the phylogenetic perspective, the Australian fauna is composed of two distinct groups: the Onthophagini, which contains the cosmopolitan genus *Onthophagus* (∼250 Australian spp. and over 2,000 spp. worldwide) that dispersed into Australia from Asia around 20 to 24 million years ago (Mya) (28), and the Australian endemic genera (AuEG) (∼250 spp.), which are a relictual Gondwanan lineage, with mid-Cretaceous origins (∼80 Mya) (29). The Australian dung beetles present a useful test case for dietary specialization and gut microbiota because a number of species in both groups have broadened their diet (30–33). In addition, there are distinct differences in behaviors and feeding strategies. The *Onthophagus* species tunnel directly beneath their food source (paracoprids), where they mass provision for their offspring, lay eggs, and then leave, engaging in little parental care (34). In contrast, the AuEG are telecoprids: i.e., they transport their food away from the sources to avoid competition, lay fewer eggs, and exhibit higher levels of parental care.

The genus *Cephalodesmius* stands out among the AuEG as having undergone the most extreme dietary shift, together with associated food processing behaviors and a high level of parental care. Males and females pair bond and work together to gather a range of organic materials, including leaves, flowers, fruit, and fungi, which are worked into a composting brood mass, thus creating a dung substitute with which to feed their larvae. The adults continue to progressively provision their larvae throughout their development to the pupal stage, exhibiting a level of subsocial behavior (33). In addition, the anterior hindgut of adult *Cephalodesmius* beetles is dilated into a “large, thin-walled, sac-like structure” that is proposed to be a fermentation chamber, a novel gut structure for an adult dung beetle, which may be capable of housing symbiotic bacteria (35). Given their subsocial behavior and the presence of a putative fermentation chamber, we hypothesized that members of the genus *Cephalodesmius* would possess a diverse and stable gut microbiota that would support their unusual food processing and brood care behavior. This specialized gut structure has not been noted in any other adult dung beetle.

Here, we examined the hindgut microbiota found in 21 Australian dung beetle species from the genera *Onthophagus* and *Cephalodesmius* and seven other Australian endemic genera (AuEG) that show different dietary adaptations. We focused on the microbial communities found in the anterior hindgut as this region of the gut provides the most suitable habitat for microbes and often has the largest microbial populations (36). Dung beetles that utilize a single food resource had a simple core gut microbial community, but in dung beetle species that pursue a greater range of food resources, we found an increasingly more complex microbial community. The gut community was strikingly different in the genus *Cephalodesmius*, where we discovered a persistent, distinct, and diverse community of gut microbes.

## RESULTS

### Bacterial diversity in the anterior hindgut.

We determined the microbiota composition from the hindgut of 18 individuals across seven species of *Onthophagus*, 32 individuals across three species of *Cephalodesmius*, and 30 individuals across seven genera (11 species) from the AuEG (Table 1; see Table S1 in the supplemental material). Although *Cephalodesmius* is part of the AuEG, it was considered separately because of the unusual feeding and nesting behavior of the beetles.

**TABLE 1:** Species list from a study of dung beetle gut microbiota^*a*^

Species	Taxonomic group	Presumed diet				Flightless
		Mainly dung	Mainly mushroom	Mixed	Unknown	
*Amphistomus* NSW1	AuEG	x				No
Cephalodesmius armiger	AuEG			x		Yes
Cephalodesmius laticollis	AuEG			x		Yes
Cephalodesmius quadridens	AuEG			x		Yes
Demarziella intermedius	AuEG	x				No
Demarziella scarpensis	AuEG	x				No
Diorygopyx simpliciclunis	AuEG			x		Yes
Diorygopyx tibialis	AuEG			x		Yes
Labroma umbratilis	AuEG				x	Yes
Lepanus australis	AuEG			x		No
*Lepanus* NSW2	AuEG			x		No
Lepanus ustulatus	AuEG			x		No
Mentophilus hollandiae	AuEG				x	Yes
Onthophagus arrilla	Onthophagini	x				No
*Onthophagus* CQ2	Onthophagini	x				No
Onthophagus dunningi	Onthophagini		x			No
Onthophagus fuliginosus	Onthophagini	x				No
Onthophagus granulatus	Onthophagini	x				No
Onthophagus kumbaingeri	Onthophagini		x			No
Onthophagus pugnax	Onthophagini	x				No
Tesserodon pilicrepus	AuEG				x	Yes

a Species are listed alphabetically with their taxonomic group, known dietary information, and whether they are flightless or not. Dietary information was compiled from Matthews (31, 32) and Ebert et al. (30), as well as unpublished data from G. B. Monteith. Undescribed species were recorded according to the nomenclature coding system devised by Geoff Monteith (Queensland Museum) and Tom Weir (Australian National Insect Collection) for Australian museum collections.

**FIG 1:**
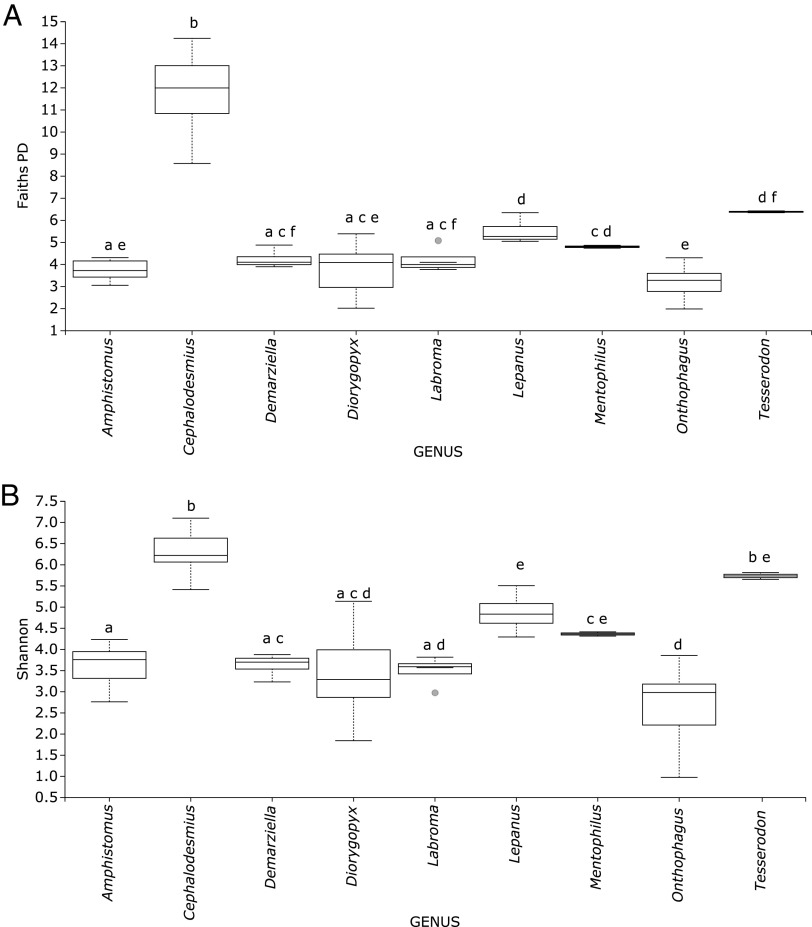
Comparisons of gut microbial community diversity within nine dung beetle genera. Box plots show diversity measured by (A) Faith’s phylogenetic diversity (PD) and (B) Shannon’s diversity index. Kruskal-Wallis tests showed a significant difference in means between genera for both measures of diversity (Shannon’s *H* = 65.4, *P* < 0.0001; Faith’s PD *H* = 65.9, *P* < 0.0001). *Cephalodesmius* shows the highest gut community diversity, and *Onthophagus* shows the lowest community diversity.

The diversity of the hindgut bacteria within each of the 9 dung beetle genera was compared using three indices: (i) Shannon’s diversity, *H*, which measures species richness (numbers of distinct taxa) and evenness (similarity of abundance); (ii) Faith’s phylogenetic diversity (PD), which incorporates phylogenetic relationships to provide an evolutionary measure of biodiversity; and (iii) evenness (pielou_e), which measures the similarity of abundance of the different taxa (distinct sequences) within the gut community (Table 2). The *Cephalodesmius* gut community consistently had the highest measures of diversity using all three indices, with more than 600 different bacterial taxa present. In contrast, the gut community of each of the other genera had fewer than 200 bacterial taxa present. The lowest-diversity measures using all indices were seen in members of the *Onthophagus*, which had fewer than 100 different bacterial taxa in the gut. A Kruskal-Wallis test showed that the mean values for both Shannon’s diversity and Faith’s PD were significantly different between the genera (Fig. 1) (Shannon’s *H* = 65.4, *P* < 0.0001; Faith’s PD *H* = 65.9, *P* < 0.0001). Evenness values ranged from 0.5 to 0.8, with the mean evenness value for *Onthophagus* being 0.59 ± 0.11: for *Cephalodesmius*, it was 0.8 ± 0, and for the remaining AuEG, it was 0.71 ± 0.08 (Table 2). A higher value of evenness (values between 0 and 1) indicates that the bacterial community is more evenly distributed; a lower value of evenness, such as was seen in *Onthophagus*, indicates that some bacterial taxa are more dominant.

**TABLE 2:** Diversity of hindgut bacterial communities in 21 Australian native dung beetle species^*a*^

Species	*n*	Total no. of sequences	Median no. of sequences/indvidual	Total no. of bacterial taxa	Diversity index value		
					Shannon’s *H*	Faith’s PD *H*	Evenness (pielou_e)
Cephalodesmius armiger	19	636,465	30,187	967	6.2	11.9	0.8
Cephalodesmius laticollis	4	132,798	34,882	640	6.5	11.8	0.8
Cephalodesmius quadridens	9	219,842	24,578	700	6.4	10.9	0.8
Tesserodon pilicripus	2	73,390	36,695	127	5.7	6.1	0.8
Mentophilus hollandiae	2	29,378	14,689	77	4.4	4.6	0.8
Labroma umbratilis	4	35,919	8,627	110	3.4	4.0	0.6
Lepanus australis	1	13,135	13,135	64	4.6	5.6	0.7
*Lepanus* NSW2	2	23,407	11,703	93	4.9	5.2	0.8
Lepanus ustulatus	2	19,851	9,925	91	4.9	5.5	0.8
*Amphistomus* NSW1	6	74,748	12,124	123	3.6	3.6	0.7
Demarziella interrupta	2	29,511	14,755	85	3.5	4.5	0.6
Demarziella scarpensis	2	22,643	11,321	74	3.8	4.2	0.7
Diorygopyx tibialis	3	19,756	6,112	91	3.7	3.8	0.7
Diorygopyx simpliciclunis	4	15,275	3,636	66	3.1	3.7	0.6
Onthophagus arrilla	1	15,839	15,839	23	3.8	3.0	0.8
*Onthophagus* CQ2	1	11,870	11,870	32	3.1	3.1	0.6
Onthophagus pugnax	4	21,832	5,513	84	2.4	3.1	0.5
Onthophagus fuliginosus	2	25,856	12,928	71	2.6	3.0	0.5
Onthophagus granulatus	4	32,547	8,097	78	2.2	2.9	0.5
Onthophagus dunningi^*b*^	5	73,517	18,743	92	3.1	3.6	0.6
Onthophagus kumbaingeri^*b*^	1	16,304	16,304	49	3.3	3.4	0.6

a *n* represents the number of samples. The total number of sequences found in each species is followed by the median number of sequences per individual. The number of taxa represents the different bacterial taxa (OTUs) found in each beetle species. Larger values of diversity indicate a greater measure of richness and evenness (Shannon’s diversity [*H*]) or phylogenetic richness (Faith’s PD). Kruskal-Wallis tests showed a significant difference in means between genera for both measures of diversity (Shannon’s *H* = 65.4, *P* < 0.0001; Faith’s PD *H* = 65.9, *P* < 0.0001). *Cephalodesmius* shows the highest gut community diversity, and *Onthophagus* shows the lowest community diversity. A higher value of evenness (values between 0 and 1) indicates that the bacterial community is more evenly distributed; a lower value of evenness indicates that some species are more dominant.

b Mushroom specialist species.

**FIG 2:**
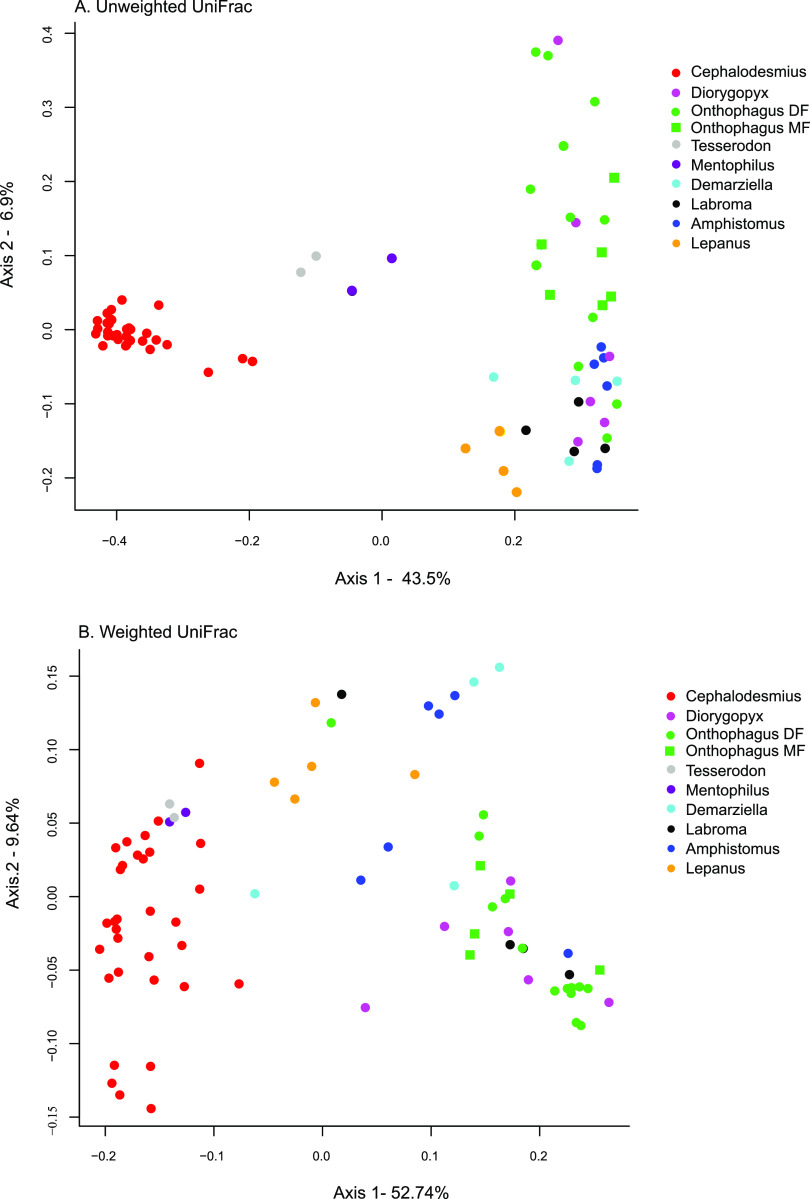
Comparisons of gut microbial community diversity between nine genera of dung beetles. β diversity measures were compared using a principal-coordinate analysis of unweighted UniFrac (A) and weighted UniFrac (B) distances. Each point represents an individual beetle sample. Genera are designated by different colors. Species in the genus *Onthophagus* are designated separately as mushroom feeders (MF) or dung feeders (DF). Proportion of variance for each axis is denoted by the corresponding axis label.

Comparisons of gut bacterial communities between beetle samples were made using unweighted and weighted UniFrac measures of diversity: the unweighted analysis takes into account the presence or absence of a particular bacterial sequence (operational taxonomic unit [OTU]) and its phylogenetic relationship to other bacterial sequences, while the weighted analysis adds the abundance of the bacterial sequences (OTUs) to the analysis. A principal-coordinate analysis (PCoA) based on unweighted and weighted UniFrac distances (Fig. 2A and B, respectively) was used to visualize comparisons of bacterial community composition between beetle genera. Bacterial communities of all individuals across three species of *Cephalodesmius* formed a distinct cluster, indicating that they shared similar gut microbiota that differed from all the other genera (Fig. 2). In contrast, *Onthophagus* gut microbiota did not cluster closely together, indicating that few taxa were shared between individuals within the genus (Fig. 2). Four genera from the AuEG had a gut community composition that overlapped *Onthophagus*, indicating some shared taxa. Two of the genera from the AuEG (*Mentophilus* and *Tesserodon*) clustered closest to *Cephalodesmius*, suggesting a similar gut composition that was distinct from the rest of AuEG and *Onthophagus*. One of the AuEG (*Lepanus*) was loosely clustered and overlapped with some of the AuEG but did not overlap *Onthophagus* in the unweighted analysis (Fig. 2A). Overall, the gut community composition of most of the AuEG appears to have more in common with *Onthophagus* than *Cephalodesmius*, with the exception of *Mentophilus* and *Tesserodon*.

### Comparisons of gut bacterial community composition between dung beetle genera.

**FIG 3:**
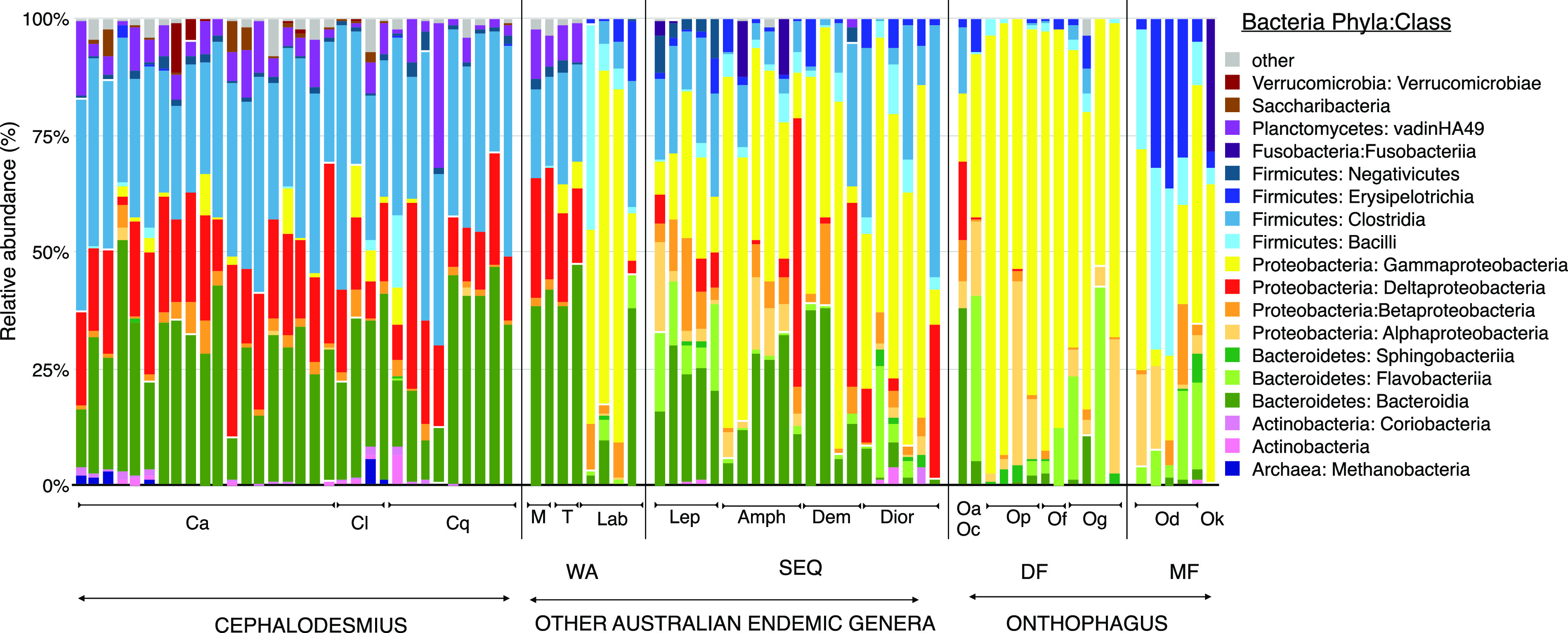
Relative abundance of bacteria (shown as phylum: class) and archaea in dung beetle gut samples. The bacterial classes are color coded by phyla, such that shades of green represent *Bacteroidetes*, yellow-orange-red are *Proteobacteria* classes, and shades of blue represent *Firmicutes*. Each bar represents a beetle sample. Members of the beetle genus *Cephalodesmius* are on the left (Ca, *Cephalodesmius armige*r; Cl, *C. laticollis*; Cq, *C. quadridens*). The other Australian endemic genera are separated into genera collected from Western Australia (WA) (M, *Mentophilus*; T, *Tesserodon*; Lab, *Labroma*) and southeast Queensland (SEQ) (Lep, *Lepanus*; Amph, *Amphistomus*; Dem, *Demarziella*; Dior, *Diorygopyx*). The *Onthophagus* gut community is shown on the right. DF indicates the dung-feeding *Onthophagus* group, and MF indicates the mushroom-feeding *Onthophagus* group (Oa, *O. arrilla*; Oc, *Onthophagus* CQ2; Op, *O. pugnax*; Of, *O. fuliginosus*; Og, *O. granulatus*; Od, *O. dunningi*; Ok, *O. kumbaingeri*).

**FIG 4:**
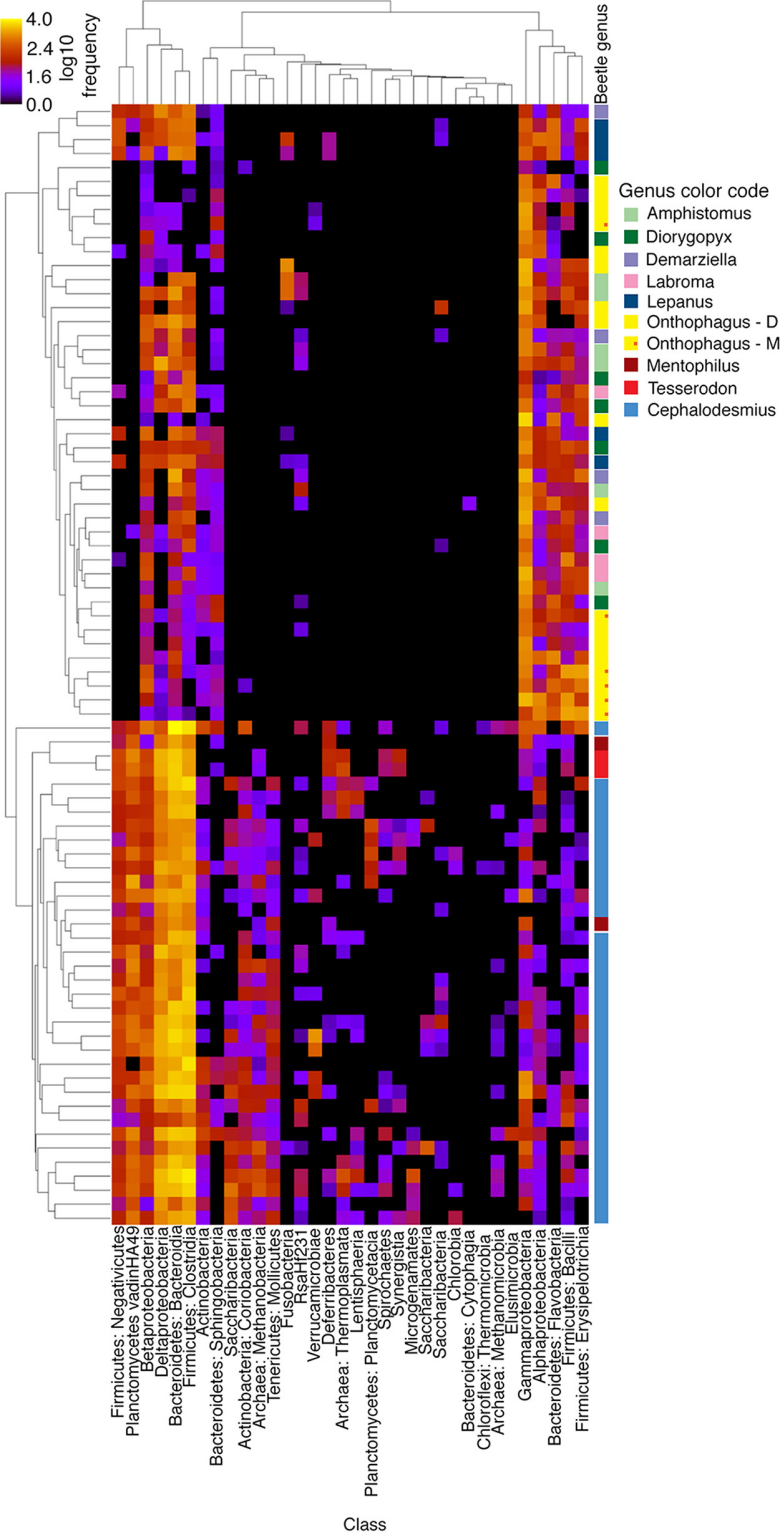
Heat map showing the composition of the gut bacterial community present in each host beetle sample. Each row represents a different beetle specimen. Beetle genera for each row are indicated by color on the right. Each column shows the presence and abundance of bacteria at the taxonomic level of class. Heat map colors indicate abundance normalized to a log scale.

Three main bacterial phyla, *Proteobacteria*, *Firmicutes*, and *Bacteroidetes*, are commonly found in various proportions in the digestive tracts of other insects and animals and are also present in all nine beetle genera considered here (Fig. 3; see Fig. S12 in the supplemental material). At the taxonomic level of bacterial class, differences between dung beetle groups become much more apparent (Fig. 3). The *Onthophagus* gut community is dominated by the class *Gammaproteobacteria* within the phylum *Proteobacteria*. The mushroom-feeding *Onthophagus* beetles also contain *Gammaproteobacteria* but have a large proportion of the classes *Bacilli* and *Erysipelotrichia* from the phylum *Firmicutes* (Fig. 3; Fig. S12). In contrast, the *Cephalodesmius* gut community is dominated by three different classes of bacteria in roughly the same proportions: *Clostridia* (in the *Firmicutes*), *Bacteroidia* (in the *Bacteroidetes*), and Deltaproteobacteria (in the *Proteobacteria*) (Fig. 3; Fig. S12). The remaining AuEG have various gut community compositions. Some of the gut communities seen in the AuEG, such as *Labroma* and *Diorygopyx*, are dominated by *Gammaproteobacteria*, similar to *Onthophagus*, but gut communities in other AuEG, such as *Mentophilus* and *Tesserodon*, share the bacterial classes of Deltaproteobacteria, *Clostridia*, and *Bacteroidia* with *Cephalodesmius* (Fig. 3). Further comparisons of the gut communities using a heat map representation of the gut taxa from each sample shows a clear relationship between the gut communities of *Cephalodesmius*, *Mentophilus*, and *Tesserodon* distinct from the other genera (Fig. 4). Traces of *Archaea* were present in only a few of the samples (Fig. 3).

### Comparisons of core bacteria of the gut microbial community at the family level.

Because bacteria make up a large part of the dung beetle diet, it is important to distinguish between bacteria that are stably associated with a taxon of beetle rather than being transient and presumably environmentally acquired. To do this, we identified core families of bacteria for each of the beetle genera (i.e., bacterial families found in over 92% of individuals in the genus). The focus on core bacterial families allowed us to make generalized comparisons between the gut microbiota of different dung beetle genera at the lowest reliable taxonomic level.

**FIG 5:**
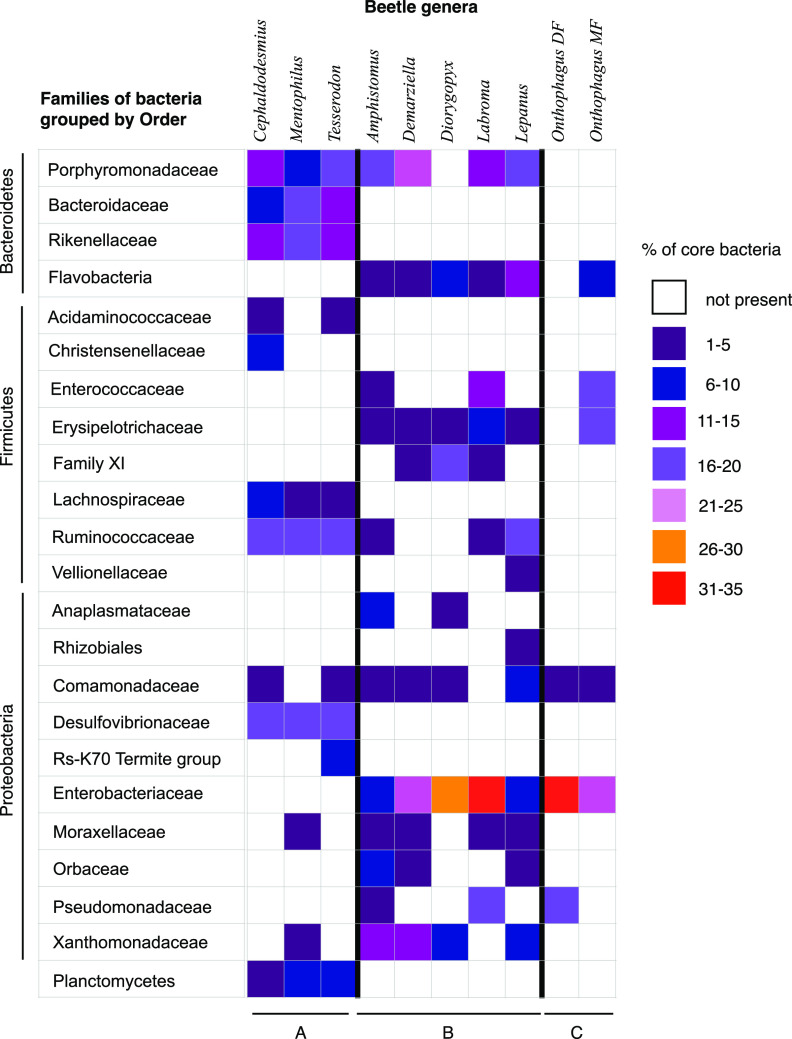
Comparisons of core bacterial families found in the hindgut of nine genera of dung beetles. Colors represent different relative proportions of the bacterial families present in each genus. Group A contains the AuEG: *Cephalodesmius*, *Mentophilus*, and *Tesserodon*. Group B contains the remaining AuEG. Group C contains the two *Onthophagus* groups: dung feeding (DF) and mushroom feeding (MF).

Thirty-two individuals from three species of *Cephalodesmius* had a consistent core gut community of 10 bacterial families regardless of the geographical location from which they were collected (Fig. 5; group A; see Fig. S3 in the supplemental material). These 10 core families made up an average of 84% of the total gut bacteria for all individuals. A large proportion of these families were anaerobic, fermentative bacteria, from the phyla *Firmicutes* and *Bacteroidetes*, including the families *Ruminococcaceae* and *Lachnospiraceae*, known to degrade complex plant material (37), and *Rikenellaceae*, which is common in fungus-cultivating termites and cockroaches and aids with the digestion of proteins (38–40). Fifteen percent of the gut bacteria were anaerobic, sulfate-reducing bacteria from the family *Desulfovibrionaceae* (*Proteobacteria*-Deltaproteobacteria), a family also found in the digestive tracts of fungus-cultivating termites, cockroaches, and humus-feeding scarab larvae (11, 38, 39). Five percent of the gut community of *Cephalodesmius* were from the phylum *Planctomycetes*, a phylum also found in detritivorous species (39, 41, 42), but not recorded in dung beetles, with the exception of a single detritivorous species that eats decaying leaves (27). Archaea were present in small amounts in some specimens but do not appear to be a consistent component of the hindgut fauna.

Within the genus *Onthophagus*, the composition of the gut community varied considerably. Only three bacterial families could be identified as core in the 12 individuals from five species of dung-feeding *Onthophagus* beetles: *Enterobacteriaceae* and *Pseudomonadaceae* (*Gammaproteobacteria*) and *Comamondaceae* (*Betaproteobacteria*) make up 55% of the total gut bacteria (Fig. 5; group C; see Fig. S10 in the supplemental material). The gut community composition of the six individuals of two species of mushroom-feeding *Onthophagus* contained five core families, including two, the *Enterobacteriaceae* and *Comamonadaceae*, that were found in the dung-feeding *Onthophagus* beetles. Additionally, two families, *Enterococcaceae* and *Erysipelotrichaceae*, from the phylum *Firmicutes* were major components of the core gut microbiota of mushroom feeders (Fig. 5; group C; see Fig. S11 in the supplemental material). Only one minor core bacterial family (*Comamonadaceae*) was shared between the core microbiota of *Onthophagus* and *Cephalodesmius* (Fig. 5).

The core gut communities of the remaining endemic genera had bacterial families that were shared with either *Onthophagus* or *Cephalodesmius* (Fig. 5; group B; see Fig. S2 to S9 in the supplemental material). The core gut communities of *Mentophilus* and *Tesserodon* were most similar to those of *Cephalodesmius*, while the remaining five endemic genera had more families in common with *Onthophagus*, especially the mushroom-feeding *Onthophagus* (Fig. 5).

Further analysis of the gut community of *Cephalodesmius* collected from widely separated geographical regions revealed that even at the level of the OTU, several taxa were abundant and present in at least 80% of all samples from the three species (Table 3). Most notable were the sulfate-reducing anaerobic *Desulfovibrio* (Deltaproteobacteria) and the fermentative, anaerobic *Clostridiales* “*Candidatus* Soleaferrea” (*Ruminococcaceae*) and *Tyzzerella* (*Lachnospiraceae*). Similar analysis of *Onthophagus* samples revealed that none of the OTUs were abundant in more than 30% of samples in the dung feeders (Table 4). However, the gut communities from two mushroom-feeding species of *Onthophagus* all shared OTUs from the *Firmicutes* family *Erysipelotrichaceae* (Table 4).

**TABLE 3:** Top five most abundant OTUs found in the hindgut of species of the dung beetle genus *Cephalodesmius*^*a*^

Taxonomy of top 5 OTUs found in 80% of samples	No. of:	
	Samples	Reads
Deltaproteobacteria		
*Desulfovibrio* sp. 1	32	59,177
*Desulfovibrio* sp. 2	31	52,519
*Bacteroidetes*		
*Rikenellaceae*: *Alistipes*	27	27,217
*Firmicutes*: *Clostridiales*		
*Ruminococcaceae*: “*Candidatus* Soleaferrea”	32	10,727
*Lachnospiraceae*: *Tyzzerella*	27	10,034

a There were three species of *Cephalodesmius* (*n* = 32 beetles). The OTUs are identified to the lowest taxonomic level available. The total number of reads was 989,105, and the total number of OTUs was 1,157.

**TABLE 4:** Top five most abundant OTUs found in the hindgut of the dung beetle genus *Onthophagus*^*a*^

*Onthophagus* group	Taxonomy of top 5 OTUs found in 30% of samples	No. of:	
		Samples	Reads
Dung feeding	*Gammaproteobacteria*		
	*Enterobacteriaceae*: *Providencia*	4	12,238
	*Enterobacteriaceae*: *Escherichia*-*Shigella*	5	1,463
	*Bacteroidetes*		
	*Flavobacteriaceae*	5	9,124
	*Alphaproteobacteria*		
	*Wolbachia*	5	3,071
	*Bacteroidetes*: *Chitinophagaceae*	9	415

Mushroom feeding	*Firmicutes*		
	*Erysipelotrichaceae*: *Erysipelothrix*	6	9,390
	*Erysipelotrichaceae*	6	6,237
	*Enterococcaceae*: *Vagococcus*	6	3,238
	*Gammaproteobacteria*		
	*Enterobacteriaceae*: *Morganella*	6	1,672
	*Bacteroidetes*: *Chitinophagaceae*	6	433

a In the dung-feeding *Onthophagus* beetles (*n* = 13), there were no abundant OTUs common to more than 9 of the samples: most OTUs occurred in 30% of samples. There were 118,046 reads and 230 OTUs total. In the mushroom-feeding *Onthophagus* beetles (*n* = 6), the five most abundant OTUs were found in all six samples. There were 89,892 reads and 128 OTUs. OTUs are identified to the lowest taxonomic level available.

### Hindgut morphology comparisons.

**FIG 6:**
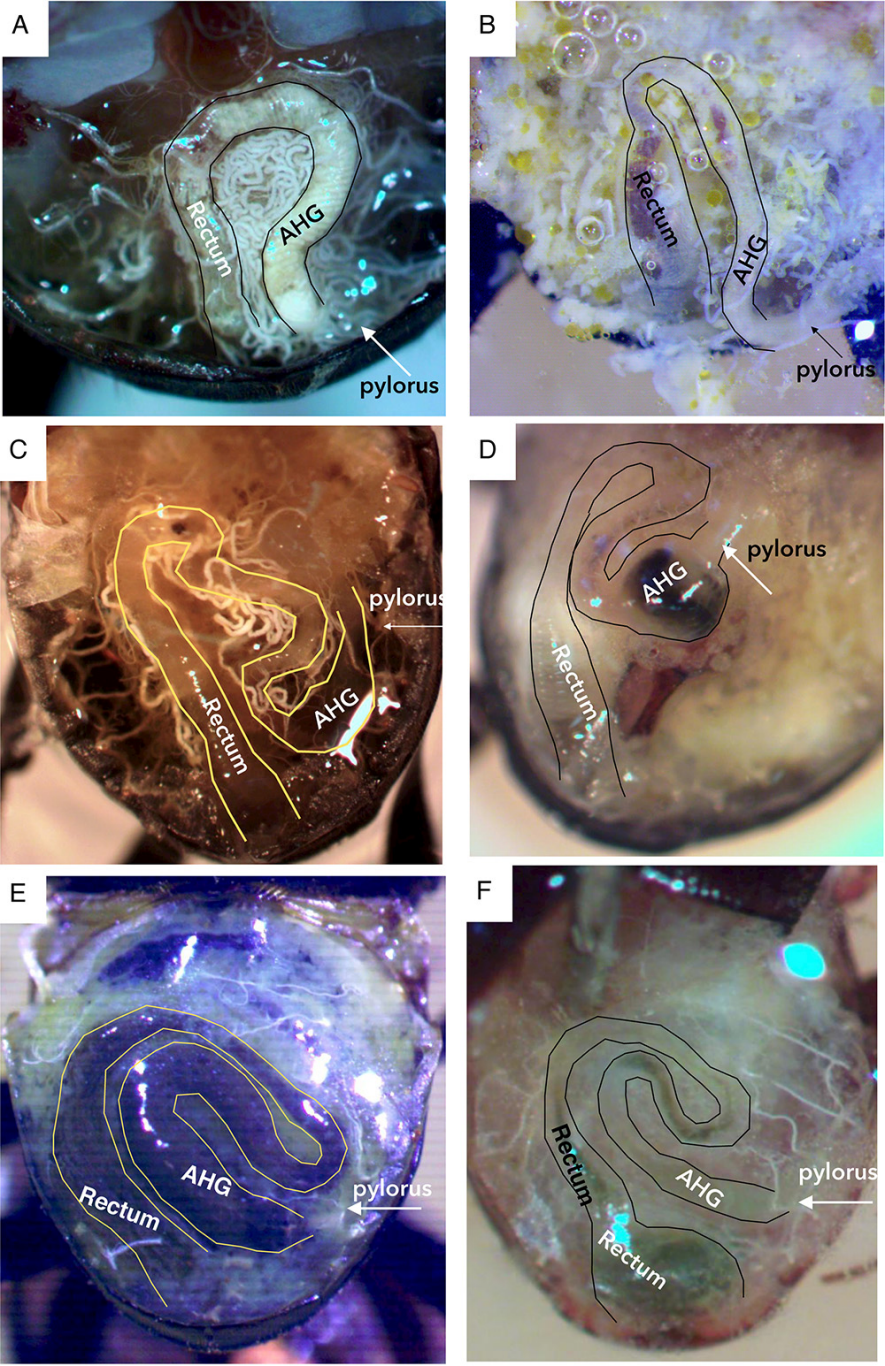
A dorsal view of *in situ* gross hindgut morphologies seen in six representative adult dung beetle species. (A) Onthophagus pugnax. The hindgut is short, with only a single U-shaped bend, and it does not have an enlarged anterior hindgut (AHG). (B) *Amphistomus* NSW1 also has a single U-shaped bend and a narrow anterior hindgut region. (C) Mentophilus hollandiae has a short, dilated region just after the pylorus, followed by a slight clockwise loop, which then loops anticlockwise. (D) Tesserodon pilicrepus also shows a short, dilated region in the anterior hindgut. (E) Cephalodesmius quadridens. From the pylorus, the anterior hindgut dilates, then it loops in a clockwise direction before contracting and reversing to an anticlockwise loop, which dilates again and terminates with the rectum. (F) *Lepanus* NSW2 hindgut shows an anterior hindgut looped clockwise first, then looped anticlockwise, without dilation in the anterior hindgut. The pylorus marks the beginning of the hindgut.

The observed differences in microbial communities might be anticipated to coincide with differences in gut structure if features of the gut were associated with unique functions of the microbiota. Two primary and two intermediate hindgut morphologies were observed in the 21 dung beetle species in this study (Fig. 6; Fig. S2 to S11), and these differences did indeed coincide with composition of the microbiota. The first was characteristic of the genus *Onthophagus*. It consisted of a short hindgut configured in a simple U-shape bend starting from the pylorus (valve between midgut and hindgut), with no dilation of the anterior region (Fig. 6A and B). The second form was characteristic of the genus *Cephalodesmius*. In the second form, the hindgut was lengthened about 2-fold to form two loops, with a dilation of the anterior region of the hindgut just after the pylorus (Fig. 6E). Intermediate forms exhibited lengthening but no dilation of the anterior region (Fig. 6F) or dilation of the anterior hindgut, but less lengthening (Fig. 6C and D). Differences in hindgut morphology coincided with differences in gut microbiota. The beetle genera with dilated anterior hindguts (*Cephalodesmius*, *Mentophilus*, and *Tesserodon*) shared a distinctly different microbiota containing Deltaproteobacteria and *Planctomycetes*, while those beetle genera with short, nondilated hindguts were dominated by *Gammaproteobacteria*.

## DISCUSSION

Australian dung beetles provide an opportunity to explore the adaptive potential of gut microbiota due to their phylogenetic diversification and different feeding strategies, which include dietary diversification and elaborate food processing behavior, as well as distinctive hindgut morphologies. Each of these factors has the potential to significantly influence the gut microbial community and, in turn, be influenced by the resident gut microbes, providing an opportunity to identify potential associations.

An important indication of whether or not the microbiota contributes to the evolution of their host is whether the composition of the microbiota follows the evolutionary trajectory of the host. It is clear from our results that the microbiota of three genera of the AuEG, most notably *Cephalodesmius*, are distinct from the microbiota of phylogenetically distinct Australian *Onthophagus* species, while the remaining members of AuEG exhibit some commonalities. The differences in microbiota within the AuEG may be related to their phylogenetic relationships. Tarasov and Genier (43) have proposed that the Australian endemic genera should be divided into two separate clades. *Cephalodemius* and *Mentophilus* are considered to be in a separate clade from several of the other AuEG, suggesting a phylogenetic component to the differences in microbiota.

An important caveat is that an apparent difference in microbiota between distinct taxa could simply relate to the location from which the insects were sampled. For *Onthophagus*, this appeared to be the case, as the composition of the microbial gut community was quite variable even between beetles of the same species. Environmental acquisition of microbial gut fauna has been noted in *Onthophagus* by others and may contribute to its adaptability (5). *Onthophagus* is a cosmopolitan genus that readily disperses to new environments (28, 44). It has been suggested that animals that are not dependent upon gut symbionts may be more able to switch to new habitats or food resources (45). The cosmopolitan distribution of *Onthophagus* species provides evidence for their adaptability, as does the fact that some have shifted from coprophagy to diets of carrion, fungi, or fruits in some habitats (29, 31, 46–50).

The situation within the genus *Cephalodesmius*, however, is quite different. Rather than having the variability of the microbiota seen within *Onthophagus*, *Cephalodesmius* species share a conserved gut microbiota of 10 core bacterial families, whereas *Onthophagus* has only two. Even at the level of the OTU, several bacterial taxa are conserved across all three species of *Cephalodesmius*. The conservation of the core microbiota in *Cephalodesmius* species is particularly remarkable in that the gut community composition is conserved across isolated populations in disjunct patches of remnant rainforest. The fact that the core microbiota is stable across geographic regions despite *Cephalodesmius* beetles being flightless suggests that the core microbiota has been stable for a long time. This stability of the association between the insect host and its microbiota is indicative of a mutually beneficial relationship that is under stabilizing selection.

An obvious possibility is that the microbiota might simply correlate with diet. Superficially, it would seem that food choice and microbiota are related as *Onthophagus* and *Cephalodesmius* exhibit the most extreme dietary preferences and also have the most divergent microbiota, with the remaining AuEG having intermediate food preferences and microbiota. We find, however, that specific food preferences are not reflected in the overall composition of the gut microbial community or in specific gut microbe taxa. Almost all of the dung beetles sampled are attracted to dung, yet some are attracted to a wider variety of non-dung food sources. Those species that feed mainly on dung did not share a consistent gut microbiota. Similarly, dung beetles with broader, varied diets did not share a consistent gut microbiota either, although there are some common taxa. For example, both *Lepanus* and *Cephalodesmius* species have been collected at a variety of different types of bait (30), yet the two genera had different gut communities. This indicates that other factors, in addition to diet, influence the gut community composition.

There is another intriguing possibility: that a relationship between diet and gut microbiota of dung beetles does exist, but that it relates to the way food is processed and the brood are fed. *Onthophagus* beetles are opportunistic, strong flyers that locate and exploit ephemeral, fresh dung resources. They dig tunnels beneath a dung source, where the female deposits dung provisions for her brood, lays eggs, then moves on to other fresh dung resources, thus producing a large number of offspring but engaging in minimal parental care (34). In contrast, *Cephalodesmius* beetles are flightless and inhabit remnant rainforests. Consistent with their isolation and restricted mobility, members of the genus *Cephalodesmius* rely on dependable, local food resources, which may, however, require processing to improve the nutritional value. The food processing activity of *Cephalodesmius* species involves a pair-bonded male and female, working together to gather dung, carrion, fungi, leaves, fruits, or flowers into a permanent nest burrow, where it is manipulated into a ball of composting material called a brood mass (33). Adult feces are added to this material, in essence inoculating the brood mass with hindgut microbiota. Both parents maintain the brood mass throughout the development of their offspring in order to provide continuous provisions. Not as much is known about the nesting behavior of the other AuEG, but they, like *Cephalodesmius* species, have low fecundity and transfer food resources to a new location away from the source (telecoprids), in contrast to *Onthophagus* species, which have high fecundity and bury the food resource at its source (paracoprids).

This study revealed that the gut community of *Cephalodesmius* beetles has more in common with gut communities of insect detritivores than with that of other coprophagic dung beetles. In a meta-analysis of insect gut communities, the most basal split in community composition separated detritivores and xylophages (dead wood feeding) from all other dietary guilds (2). The detritivore gut microbiota is distinctive, dominated by *Clostridiales*, *Bacteroidales*, and Deltaproteobacteria—the same classes of bacteria we see in *Cephalodesmius*. These groups form only minor components in the gut community of nondetritivorous insect diet guilds (2). The higher termites comprise much of the detritivore guild (51); however, convergences to the same gut community structure are seen in other detritivores, such as humus-feeding scarab larvae (11) and detritus-feeding fly larvae (15). Subsequently, gut microbiota studies of a number of omnivorous insects have revealed a picture of different degrees of convergence to this detritivore-type microbiota: they include field crickets (10), the New Zealand weta (Orthoptera) (42), and cockroaches (39). An emerging picture is that detritivores tend to converge on a similar gut community, but each major taxonomic group has unique components.

A common anatomical feature shared between *Cephalodesmius* and other detritivores is the expanded hindgut, which appears to have a significant impact on the composition and function of the gut microbiota. Similar gut alterations have never been observed in *Onthophagus*, providing support for the notion that the structural changes may be closely tied to the retention of the distinctive *Cephalodesmius* microbiota across evolutionary time and geographical distance. It is interesting to note that members of two other genera in the AuEG, *Mentophilus* and *Tesserodon*, have microbiota very similar to that of *Cephalodesmius* species, as well as a degree of hindgut dilation. The detritivore-type microbial community, the diverse and stable core microbiota, and corresponding gut dilation in the three beetle genera *Cephalodesmius*, *Mentophilus*, and *Tesserodon* support the hypothesis that the expanded anterior hindgut is functioning as a fermentation chamber that houses symbiotic bacteria to assist with digestion. The distinctive digestive system may have provided an essential microhabitat for the establishment of environmental bacteria in the gut, therefore allowing *Cephalodesmius* to exploit a new behavioral niche.

The most abundant OTUs common to all the *Cephalodesmius* samples were *Desulfovibrio* in the Deltaproteobacteria. *Desulfovibrio* has also been found to be abundant in the hindgut of larvae in other scarab subfamilies (17, 18). In *Melolontha* larvae, *Desulfovibrio* species specifically colonize the hindgut wall, while many of other gut bacteria are restricted to the gut lumen, suggesting that the sulfate-reducing *Desulfovibrio* species are adapted to colonize this microhabitat (18). It would seem plausible that environmentally acquired *Desulfovibrio* could be preferentially selected and adapted to live in this particular gut microhabitat in adult *Cephalodesmius* beetles, similar to what has been documented in cockroaches (52).

Why do *Cephalodesmius*, *Mentophilus*, and *Tesserodon* beetles share a core community that more closely resembles that of detritivores such as cockroaches than it does that of pure dung feeders such as members of the *Onthophagus*? The community likeness does not appear to be habitat based because while *Cephalodesmius* beetles live in rainforest areas in eastern Australia, *Mentophilus* and *Tesserodon* beetles are found in arid, open regions along the dry central coastal regions of Western Australia. The only behavioral observations of *Mentophilus* and *Tesserodon* beetles indicate that they bury old, dried fecal pellets deep in the ground below the moisture line (32), where the moisture may revive bacterial and fungal activity in the fecal pellets, thus providing a microbial food source for the beetles or their larvae. It is possible that feeding on once-dried fecal pellets necessitates a core gut microbiota more typical of detritus feeders, and *Cephalodesmius* beetles’ feeding on composting organic matter places the same demands. It has also been noted that *Mentophilus* beetles have been found frequently under mushrooms and once feeding on dead beetle larvae (32). A closer examination of *Mentophilus* and *Tesserodon* beetle behavior is warranted to establish whether their diet includes additional detritus components or even possibly fungus cultivation.

In beetles from the *Cephalodesmius* genus, we suspect that the detritus-associated microbiota is associated with the fact that the brood ball materials they consume are made of composted plant and organic matter, fed upon and added to over time. This is evidenced by the abundance of plant-degrading bacteria in their gut community. Also, the filtering mandibles used by adult dung feeders to obtain nutrients from fresh dung might not be as effective when compost is the food source, leading to a requirement for detritivore-style bacterial community and a gut fermentation chamber. A point to be borne in mind is that the diet and, consequently, the gut microbiota of the adult gut might also be related to larval nutrition. Larvae are reliant on nutrients in the brood ball, and consequently, the adults might carry a microbiota whose substantial role is to facilitate larval nutrition, as reported for members of the dung beetle genus *Euoniticellus* (26).

The question arises that since dung beetles arose from detritivorous ancestors (53), are the diet and gut microbiota found in *Cephalodesmius* species an ancestral condition? Monteith and Storey (33) suggest that the advanced food processing and complex subsocial behaviors seen in *Cephalodesmius* beetles are highly specialized rather than ancestral. To understand the evolution of these gut microbial communities, it would be useful to examine the gut microbiota in other dung beetles that are closely related to members of the *Cephalodesmius*, in addition to more primitive beetle relatives, such as those in Geotrupidae. Examination of the gut microbiota of other dung beetles from the two different clades of AuEG may provide further evidence of phylogenetic associations in gut community composition.

### Conclusions.

Overall, this study has revealed an unexpected diversity in the gut microbiota of dung beetles, which may have facilitated the adaptation and evolution required to expand into new habitats or new behaviors. This is most apparent in the dichotomy between *Onthophagus* and *Cephalodesmius* species. Our findings suggest that a stable evolutionary partnership between members of the genus *Cephalodesmius* and its highly conserved gut microbiota may have allowed the exploitation of abundant, but low-quality, non-dung food resources. The strongly conserved microbiota across evolutionary time and geographical isolation indicates a coevolved, mutually beneficial association. The distinctive morphology of the gut of *Cephalodesmius* beetles may provide a fermentation chamber that facilitates the function of the microbiota or that ensures that essential microbes are retained. It seems likely that the microbiota has helped members of the *Cephalodesmius* to adapt to a niche where dung is less abundant, leading to the coevolution of behaviors required for the processing of alternative food resources. The time and effort required to process the food items that are collected necessitates the continuous provisioning of the brood, resulting in extended biparental care. Clearly, multiple factors affect the composition of gut microbiota, and further examination of gut microbial communities will add to the overall understanding of the evolutionary influence of gut microbiota.

## MATERIALS AND METHODS

### Beetle collection.

A total of 81 dung beetle specimens from nine genera and 21 species were collected from southeast Queensland and Western Australia between 2016 and 2018 (see Fig. S1 and Table S1 in the supplemental material). Beetle species were selected to represent different phylogenetic groups (Onthophagini and Australian endemic genera [AuEG]) as well as different diet groups. Beetles were collected mainly from southeast Queensland in areas where sampling has been done extensively in the past so that the dung beetles could be readily identified. Western Australian species were included in order to have additional species from a different biogeographical region. Of the 21 species, seven were from *Onthophagus*, three were from *Cephalodesmius*, and 11 were from other AuEG (Table S1). Sampling was particularly focused on areas where *Cephalodesmius* beetles occur, since this was a genus of particular interest.

Beetles were collected alive: either from pitfall traps baited with kangaroo (Macropus giganteus) dung or rotting mushroom or directly excavated from nest burrows. Specimens were identified to the species level. Undescribed species were recorded according to the nomenclature coding system devised by Geoff Monteith (Queensland Museum, Brisbane, QLD) and Tom Weir (Australian National Insect Collection, Canberra, ACT) for Australian museum collections. Voucher specimens are stored at the Queensland Museum.

### Dissections.

After collection, beetles were housed for 4 to 5 days in plastic containers without food prior to dissections. Beetles were euthanized by being placed in a freezer (–20°C) for at least 1 h and then were dissected immediately upon removal. All dissection equipment was sterilized in 30% sodium hypochlorite solution and then rinsed in distilled water. Beetles were surface sterilized by immersion in 75% ethanol for 1 min prior to dissection. The dissections took place in three stages: (i) removal of elytra, (ii) removal of dorsal abdominal cuticle, with the hindgut photographed *in situ*, and then (iii) removal of anterior hindgut (starting from the end of the narrow pyloric valve to the first bend of the hindgut). Tools were sterilized between each stage to limit contamination of the final sample.

Prior to the removal of the anterior hindgut from the abdominal cavity, the entire hindgut was photographed *in situ* for each species using a Touptek USB microscope camera and Touplite photographic software for MacOS.

### DNA extraction.

DNA was extracted from each sample using the HotSHOT DNA extraction procedure (54). Hindgut samples were immersed in alkaline lysis reagent (25 μM NaOH, 0.2 μM EDTA [pH 12]), mashed with a sterile pipette tip, heated to 95°C for 30 min, and then neutralized with Tris-HCl (40 μM). Samples were stored at –20°C until use.

### DNA sequencing and taxon classification.

Amplicon sequencing of 16S rRNA genes was performed by the Australian Centre for Ecogenomics (ACE) (University of Queensland, St. Lucia, QLD, Australia) using the 803F forward primer (5′-TTAGAKACCCBNGTAGTC-3′) and the 1392wR reverse primer (5′-ACGGGCGGTGWGTRC-3′) for the V6 to V8 region on the MiSeq sequencing system (Illumina, Inc.). All sequence data were initially processed and quality filtered through the ACE pipeline with fastQC. Sequences were trimmed to 250 bases to remove primer sequences and poor-quality sequences using Trimmomatic (55) and then processed using the DADA2 denoising algorithm (56). Taxonomic assignments for sequences were based on 97% identity and obtained from the Silva and UNITE reference databases using BLAST+ (57).

### Sequence filtering and data analysis.

All processing and analyses of microbial data were conducted using QIIME 2-2018.2 Microbiome analysis software (58). All unassigned (failed to classify) and Eukaryota sequences and any sequences not identifiable beyond domain were removed. Sequences that only occurred in one individual or had a frequency of less than 100 reads were also removed. For diversity analyses, data were rarefied to a sampling depth of 2,500 (98.8%) reads to minimize effects of uneven sequence counts between samples.

Data were analyzed using the QIIME 2 Core Metrics Phylogeny, which aligns sequences phylogenetically to produce diversity data. Bacterial community diversity within samples (α diversity) was assessed using Shannon’s and Faith’s phylogenetic diversity (PD) indices (59, 60). Effects of beetle genus on alpha diversity were assessed using Kruskal-Wallis pairwise comparisons. Bacterial community diversity between beetle samples (β diversity) was calculated using unweighted and weighted UniFrac distances to assess phylogenetic diversity. UniFrac, an abbreviation of “unique fraction,” is a phylogenetic technique developed specifically for microbial communities that measures community similarity based on the bacterial lineages they contain (61). Principal-coordinate analysis (PCoA) was used to visualize the community similarity between beetle genera. Heat maps of the gut microbial communities in each beetle specimen were also generated using QIIME2.

### Defining and analyzing core microbiota.

In order to make detailed comparisons, a core group of bacteria was identified for each genus of beetle. Since many of the bacterial sequences could not be reliably identified below the family level, we chose to look at core families, as this taxonomic level would achieve the most detail, yet still be reliable. Core families were determined to be those which were present in 100% of individuals within a given genus, with the exception of *Onthophagus* and *Cephalodesmius*. As these two genera had larger sample sizes, we defined core families as those being present in all but one individual (31 out of 32 for *Cephalodesmius* and 11 out of 12 for *Onthophagus*). The proportions of taxa comprising the core were calculated by dividing the number of core families by the total number of families. The quantitative contribution of each core bacterial family taxon was calculated as the proportion of reads assigned to each core relative to total reads for each beetle genus.

### Data availability.

All 16S rRNA sequences are accessible in the NCBI Sequence Read Archive (SRA) under accession no. PRJNA638479.

## Supplementary Material

Supplemental file 1
