# Supplementary material for: Hindgut Microbiota Reflects Different Digestive Strategies in Dung Beetles (Coleoptera: Scarabaeidae: Scarabaeinae)
Source: Appl Environ Microbiol. 2021 Feb 12;87(5):e02100-20. doi: 10.1128/AEM.02100-20 (PMC8090880; doi:10.1128/AEM.02100-20)
Supplement: Supplemental file 1 [file AEM.02100-20-s0001.pdf]

Table S1. **Species list**. Details of the dates and collecting localities for the dung beetle gut microbe community samples. Australian Endemic Genera (AuEG) species are listed in alphabetical order followed by the *Onthophagus* species in alphabetical order.

**Species**	**n**	**S**	**locality**	**state**	**coordinates**	**date collected**
Amphistomus NSW1	4	M	Lamington: O'Reillys	QLD	-28.22, 153.14	Feb 2018
Amphistomus NSW1	2	M	Springbrook: Ankida	QLD	-28.16, 153.26	Mar 2018
Cephalodesmius armiger	4	M,F	Lamington: O'Reillys	QLD	-28.22, 153.14	May 2017, Feb 2018
Cephalodesmius armiger	3	M,F	Levers Plateau	QLD	-28.29, 152.86	Jan 2018
Cephalodesmius armiger	2	M,F	Mt Glorious	QLD	-27.24, 152.78	Jan 2018
Cephalodesmius armiger	2	F	Yabbra	NSW	-28.52, 152.56	Dec 2017
Cephalodesmius armiger	2	M,F	Springbrook: Bandicoot	QLD	-28.21, 153.27	May 2017
Cephalodesmius armiger	3	M,F	Cunningham's Gap	QLD	-28.03, 152.40	Feb 2016, May 2017
Cephalodesmius armiger	3	M,F	Bunya Mtns	QLD	-26.80, 151.61	Aug 2017
Cephalodesmius laticollis	2	M,F	Springbrook: Repeater	QLD	-28.22, 153.27	May 2017
Cephalodesmius laticollis	1	M	Mt Tamborine	QLD	-27.91, 153.21	Jun 2017
Cephalodesmius laticollis	1	ND	Lamington: BinnaBurra	QLD	-28.20, 153.19	Feb 2016
Cephalodesmius quadridens	1	ND	Dwyer Scrub	QLD	-27.66, 152.11	Feb 2016
Cephalodesmius quadridens	4	M,F	Bunya Mtns	QLD	-26.80, 151.61	Aug 2017
Cephalodesmius quadridens	1	F	Boombana	QLD	-27.35, 152.82	Jan 2018
Cephalodesmius quadridens	3	M,F	Gold Creek	QLD	-27.46, 152.88	Feb 2018, Mar 2018
Demarziella interrupta	2	M,F	Lamington: O'Reillys	QLD	-28.22, 153.14	Feb 2018
Demarziella scarpensis	2	M,F	Springbrook: Ankida	QLD	-28.16, 153.26	Mar 2018
Diorygopyx simpliciclunis	3	F	Lamington: O'Reillys	QLD	-28.22, 153.14	Feb 2018
Diorygopyx tibialis	3	M,F	Springbrook: Ankida	QLD	-28.16, 153.26	Aug 2017, Mar 2018
Labroma umbratilis	4	M,F	Seabird	WA	-31.27, 115.44	Sept 2017
Lepanus australis	1	M	Gold Creek	QLD	-27.46, 152.88	Feb 2018
Lepanus NSW2	2	M,F	Springbrook: Ankida	QLD	-28.16, 153.26	Mar 2018
Lepanus ustulatus	1	F	Springbrook: Ankida	QLD	-28.16, 153.26	Mar 2018
Lepanus ustulatus	1	F	Boombana	QLD	-27.35, 152.82	Jan 2018
Mentophilus hollandiae	2	F	Quobba	WA	-24.48, 113.42	Aug 2017
Tesserodon pilicrepus	2	M	Quobba	WA	-24.48, 113.42	Aug 2017

**Species**	**n**	**S**	**locality**	**state**	**coordinates**	**date collected**
Onthophagus arrilla	1	ND	Springbrook: Ankida	QLD	-28.16, 153.26	Aug 2017
Onthophagus CQ2	1	ND	Springbrook: Ankida	QLD	-28.16, 153.26	Aug 2017
Onthophagus dunningi	4	M,F	Gold Creek	QLD	-27.46, 152.88	Mar 2018
Onthophagus dunningi	1	F	Springbrook: Ankida	QLD	-28.16, 153.26	Mar 2018
Onthophagus fuliginosus	2	M,F	Lamington: O'Reillys	QLD	-28.22, 153.14	Feb 2018
Onthophagus granulatus	4	F	Imbil	QLD	-26.47, 152.63	Mar 2018
Onthophagus kumbaingeri	1	F	Springbrook: Ankida	QLD	-28.16, 153.26	Mar 2018
Onthophagus pugnax	3	M,F	Bunya Mtns	QLD	-26.80, 151.61	Nov 2017
Onthophagus pugnax	1	F	Lamington: O'Reillys	QLD	-28.22, 153.14	Feb 2018

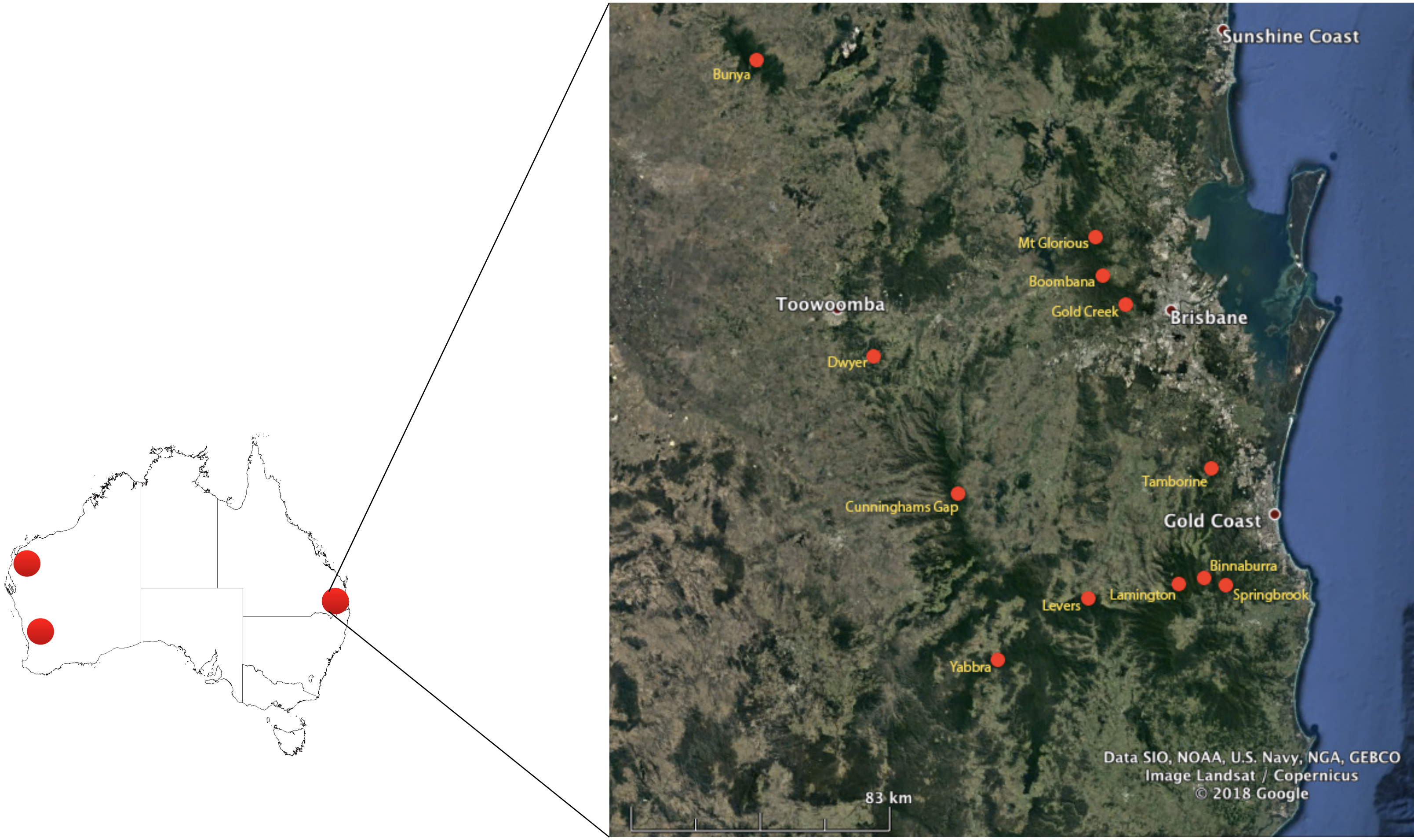

Figure S1. Map of Australia showing the three general locations where dung beetle species were collected. Expanded map (right) shows the twelve collecting sites in southeast Queensland. SE Queensland populations are distributed in rainforest areas. See Supplemental Table 1 for detailed location information. Southeast Queensland map accessed from Google Earth. Line map of Australia accessed from Wikimedia Commons (Creative Commons Attribution-Share Alike 3.0).

Table S2. Core bacterial families present in *Amphistomus*. Relative abundance of bacterial families is calculated from an average of six individuals of *Amphistomus* NSW1.

Phylum: CLASS: Family	average relative frequency	total for phylum
Bacteroidetes: BACTEROIDIA Porphyromonadaceae	19.5	20.44
Bacteroidetes: FLAVOBACTERIIA Flavobacteriaceae	0.9	
Firmicutes: BACILLI: Enterococcaceae	3.2	10.4
Firmicutes: CLOSTRIDIA: Ruminococcaceae	5.4	
Firmicutes: ERYSIPELOTRICHIA; Erysipelotrichaceae	1.8	
Proteobacteria: ALPHAPROTEOBACTERIA: Anaplasmataceae	5.9	5.9
Proteobacteria: BETAPROTEOBACTERIA: Comamonadaceae	4.1	4.1
Proteobacteria: GAMMAPROTEOBACTERIA: Enterobacteriaceae	8.3	40.2
Proteobacteria: GAMMAPROTEOBACTERIA: Orbaceae	8.0	
Proteobacteria: GAMMAPROTEOBACTERIA: Moraxellaceae	0.9	
Proteobacteria: GAMMAPROTEOBACTERIA: Pseudomonadaceae	3.7	
Proteobacteria: GAMMAPROTEOBACTERIA: Xanthomonadaceae	19.3	
total % core	81	

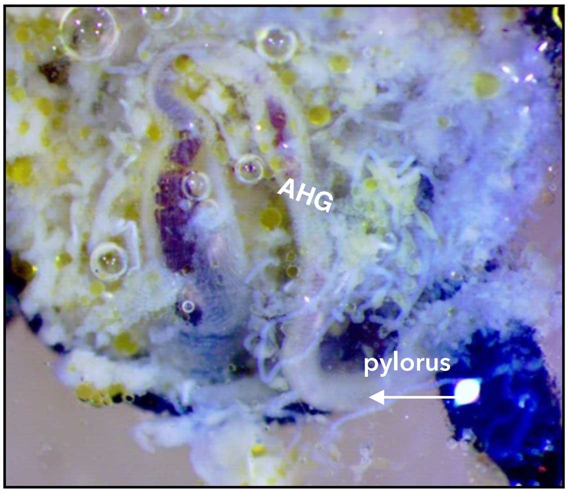

Fig.S2.*Amphistomus* hindgut. *Amphistomus* NSW1 hindgut *in situ* shows a short hind-gut and a narrow anterior section.

Table S3. Core bacterial families present in *Cephalodesmius*. Relative abundance of bacterial families is calculated from an average of 97% of individuals (31/32) from three species: *Cephalodesmius quadridens*, *C. armiger* and *C. laticollis*.

Phylum: CLASS: Family	average relative frequency	total for phylum
Actinobacteria: CORIOBACTERIIA: Coriobacteriaceae	0.5	0.5
Bacteroidetes: BACTEROIDIA Porphyromonadaceae	10.8	29.7
Bacteroidetes: BACTEROIDIA Bacteroidaceae	6.2	
Bacteroidetes: BACTEROIDIA Rikenellaceae	12.7	
Firmicutes: NEGATIVICUTES Acidaminococcaceae	1.1	33.1
Firmicutes: CLOSTRIDIA Ruminococcaceae	19.4	
Firmicutes: CLOSTRIDIA Lachnospiraceae	6.0	
Firmicutes: CLOSTRIDIA Clostridiales Family XIII	0.4	
Firmicutes: CLOSTRIDIA Christensenellacae	6.2	
Proteobacteria: BETAPROTEOBACTERIA: Oxalobacteriaceae	0.4	0.4
Proteobacteria: BETAPROTEOBACTERIA: Comamonadaceae	1.6	1.6
Proteobacteria: DELTAPROTEOBACTERIA: Desulfovibrionaceae	15.3	15.3
Planctomycetes: vadinHA49	4.8	4.8
total core	85.4	

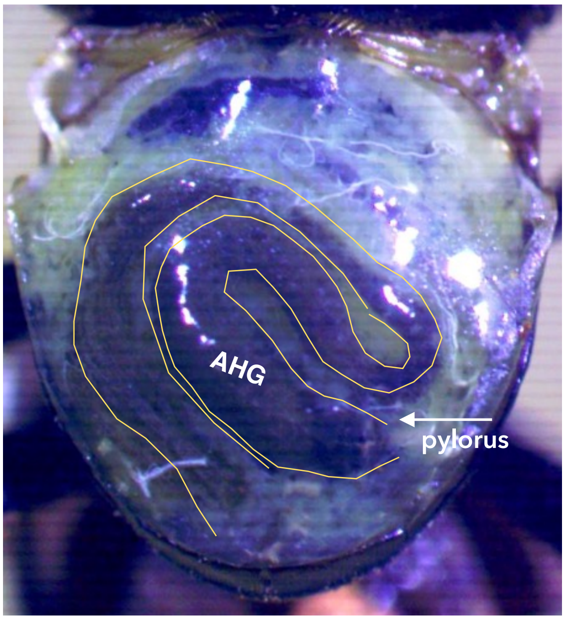

Fig. S3. *Cephalodesmius* hindgut. Hindgut of *Cephalodesmius quadridens in situ* shows the expanded anterior hindgut region and the additional gut loop to accommodate the extra length.

Table S4. Core bacterial families present in *Demarziella*. Relative abundance of bacterial families is calculated from an average of four individuals of *Demarziella:* two from each species of *Demarziella interrupta* and *D. scarpensis*.

Phylum: CLASS: Family	average relative frequency	total for phylum
Bacteroidetes: BACTEROIDIA Porphyromonadaceae	25.4	26.5
Bacteroidetes: FLAVOBACTERIIA	1.1	
Firmicutes: BACILLI: Erysipelotrichaceae	4.4	7.0
Firmicutes: CLOSTRIDIA: Family XI	2.3	
Firmicutes: BACILLI: Lactobacillales	0.3	
Proteobacteria: BETAPROTEOBACTERIA: Comamonadaceae	1.8	2.1
Proteobacteria: BETAPROTEOBACTERIA: Alcaligenaceae	0.3	
Proteobacteria: GAMMAPROTEOBACTERIA: Enterobacteriaceae	23.3	43.0
Proteobacteria: GAMMAPROTEOBACTERIA: Xanthomonadaceae	17.4	
Proteobacteria: GAMMAPROTEOBACTERIA: Moraxellaceae	1.6	
Proteobacteria: GAMMAPROTEOBACTERIA: Orbaceae	0.7	
core percentage:	78.6	

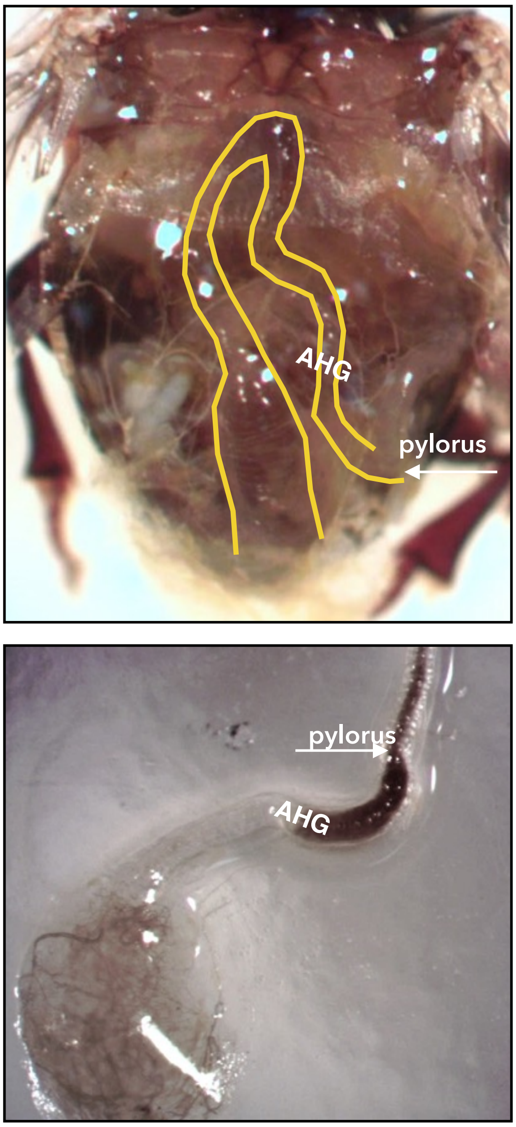

Fig.S4. *Demarziella interrupta* hindgut. Above: gut *in situ;* Below: gut removed for better viewing. Anterior hind gut is narrow and short.

Table S5. Core bacterial families present in *Diorygopyx*. Relative abundance of bacterial families is calculated from an average of six individuals of *Diorygopyx* (three from each species: *Diorygopyx tibialis* and *D. simpliciclunis*).

Phylum: CLASS: Family	average relative frequency	total for phylum
Bacteroidetes: CHITINOPHAGIA: Chitinophagaceae	0.3	6.0
Bacteroidetes: FLAVOBACTERIIA: Flavobacteriaceae	5.7	
Firmicutes: CLOSTRIDIA: Family XI	17.5	20.9
Firmicutes: BACILLI: Erysipelotrichaceae	3.4	
Proteobacteria: ALPHAPROTEOBACTERIA: Anaplasmataceae	3.3	6.1
Proteobacteria: BETAPROTEOBACTERIA: Comamonadaceae	2.8	
Proteobacteria: GAMMAPROTEOBACTERIA: Enterobacteriaceae	25.6	27.3
Proteobacteria: GAMMAPROTEOBACTERIA: Xanthomonadaceae	1.7	
total core	60.3	

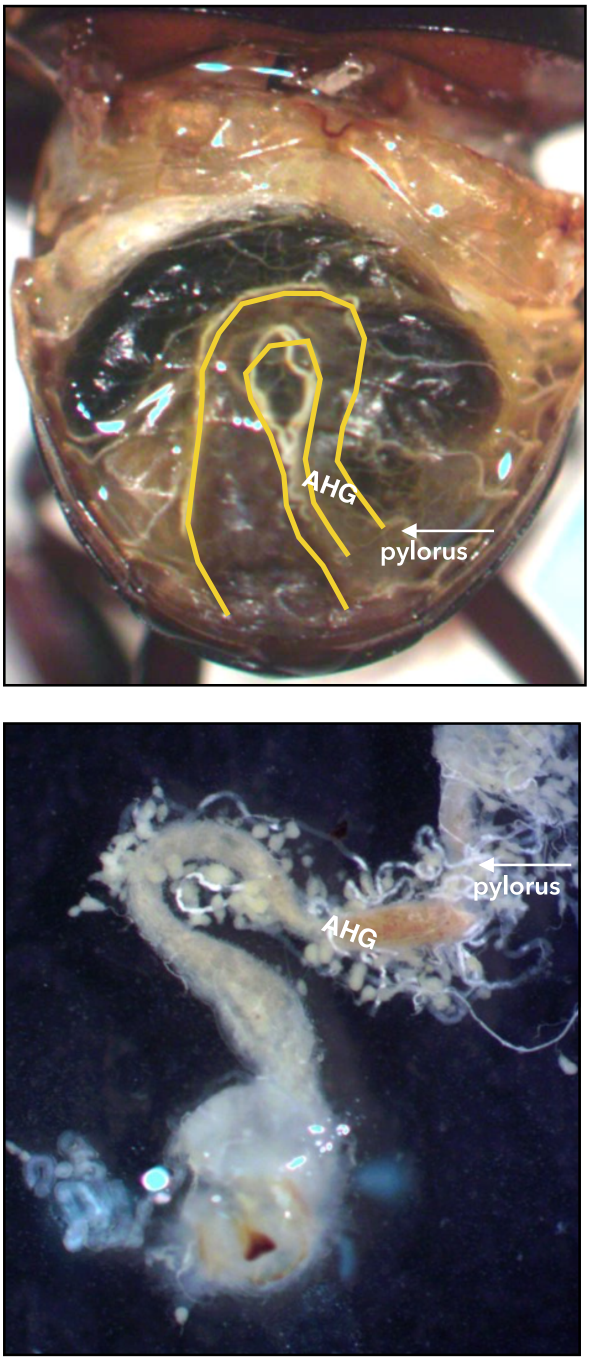

Fig.S5. *Diorygopyx* hindgut. Above: *Diorygopyx tibialis* gut *in situ*. Below: gut removed for better viewing. Anterior hind gut is narrow and short.

Table S6. Core bacterial families present in *Labroma*. Relative abundance of bacterial families is calculated from an average of four individuals of *Labroma umbratilis*.

Phylum: CLASS: Family	average relative frequency	total for phylum
Bacteroidetes: BACTEROIDIA Porphyromonadaceae	11.8	14.8
Bacteroidetes: FLAVOBACTERIIA Flavobacteriaceae	2.9	
Bacteroidetes: SPHINGOBACTERIA: Sphingobacteriaceae	0.1	
Firmicutes: CLOSTRIDIA: Family XI	5.3	27.8
Firmicutes: CLOSTRIDIA: Ruminococcaceae	4.2	
Firmicutes: BACILLI: Erysipelotrichaceae	5.5	
Firmicutes: BACILLI: Enterococcaceae	12.8	
Proteobacteria: ALPHAPROTEOBACTERIA: Anaplasmataceae	0.1	0.1
Proteobacteria: BETAPROTEOBACTERIA: Comamonadaceae	0.3	0.3
Proteobacteria: GAMMAPROTEOBACTERIA: Enterobacteriaceae	31.8	49.1
Proteobacteria: GAMMAPROTEOBACTERIA: Xanthomonadaceae	0.3	
Proteobacteria: GAMMAPROTEOBACTERIA: Pseudomonadaceae	15.9	
Proteobacteria: GAMMAPROTEOBACTERIA: Moraxellaceae	1.1	
total core	92.1	

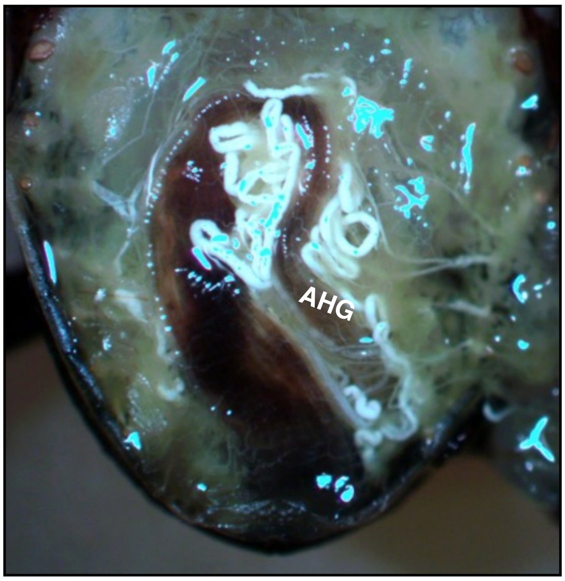

Fig.S6. *Labroma* hindgut. The *Labroma umbratilis* hindgut *in situ* shows a narrow anterior region and a short U-shaped gut.

Table S7. Core bacterial families present in *Lepanus sp.* Relative abundance of bacterial families is calculated from an average of four individuals from three species of *Lepanus*: *Lepanus ustulatus, L. australis, and L. NSW2.*

Phylum: CLASS: Family	average relative frequency	total for phylum
Bacteroidetes: BACTEROIDIA Porphyromonadaceae	20.3	33.1
Bacteroidetes: FLAVOBACTERIIA Flavobacteriaceae	12.8	
Firmicutes: CLOSTRIDIA: Ruminococcaceae	18.0	25.8
Firmicutes: BACILLI: Erysipelotrichaceae	2.5	
Firmicutes: NEGATIVICUTES: Vellionellaceae	5.3	
Proteobacteria: ALPHAPROTEOBACTERIA: Rhizobiales	1.4	1.4
Proteobacteria: BETAPROTEOBACTERIA: Comamonadaceae	7.0	7.0
Proteobacteria: GAMMAPROTEOBACTERIA: Enterobacteriaceae	6.4	14.6
Proteobacteria: GAMMAPROTEOBACTERIA: Orbaceae	1.4	
Proteobacteria: GAMMAPROTEOBACTERIA: Moraxellaceae	4.1	
Proteobacteria: GAMMAPROTEOBACTERIA: Pseudomonadaceae	0.4	
Proteobacteria: GAMMAPROTEOBACTERIA: Xanthomonadaceae	2.3	
total core:	81.9	

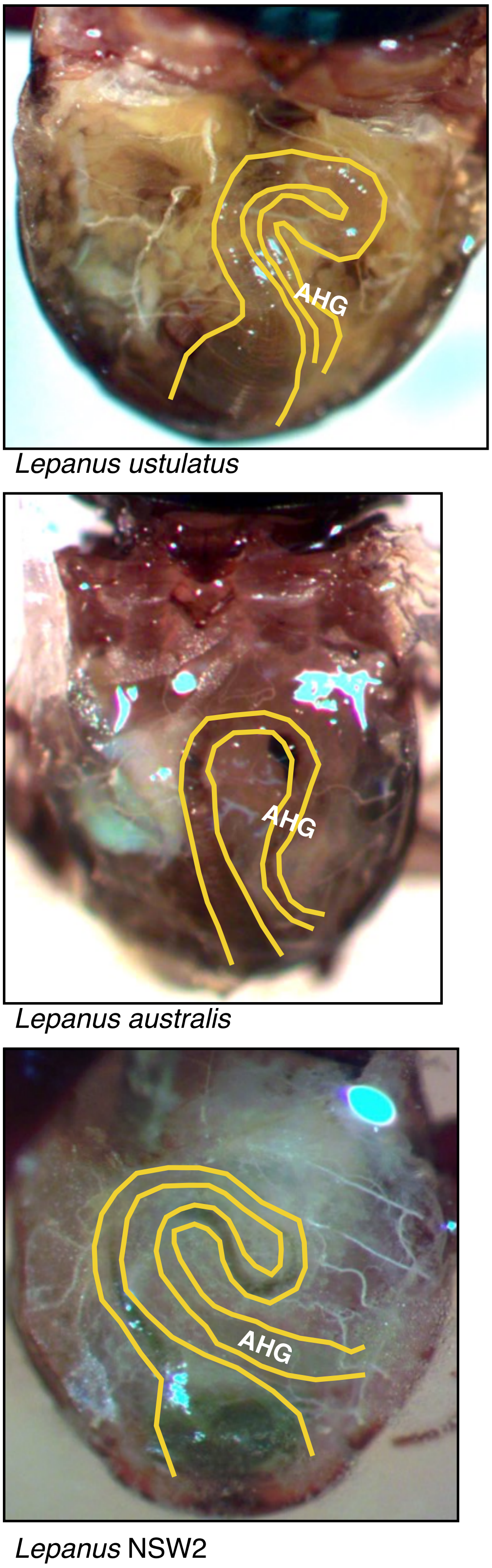

Fig.S7. *Lepanus* hindguts. Three species of *Lepanus* hindgut *in situ* showing slightly different gut configurations for each species.

Table S8. Core bacterial families present in *Mentophilus*. Relative abundance of bacterial families is calculated from an average of two individuals of *Mentophilus hollandiea*e.

**Phylum: CLASS: Family**	**average relative frequency**	**total for phyum**
Bacteroidetes: BACTEROIDIA Bacteroidaceae	15.6	43.1
Bacteroidetes: BACTEROIDIA Porphyromonadaceae	9.9	
Bacteroidetes: BACTEROIDIA Rikenellaceae	17.6	
Deferribacteres: DEFERRIBACTERES: Deferribacteraceae	0.7	0.7
Firmicutes: BACILLI: Enterococcaceae	0.1	22.7
Firmicutes: CLOSTRIDIA Christensenellaceae	0.1	
Firmicutes: CLOSTRIDIA Lachnospiraceae	4.1	
Firmicutes: CLOSTRIDIA: Peptococcaceae	0.7	
Firmicutes: CLOSTRIDIA Ruminococcaceae	17.1	
Firmicutes: NEGATIVICUTES: Vellionellaceae	0.5	
Planctomycetes: vadinHA49	8.0	8.0
Proteobacteria: DELTAPROTEOBACTERIA: Desulfovibrionaceae	17.78	17.78
Proteobacteria: GAMMAPROTEOBACTERIA: Moraxellaceae	0.75	6.1
Proteobacteria: GAMMAPROTEOBACTERIA: Xanthomonadaceae	5.31	
total	98.24	

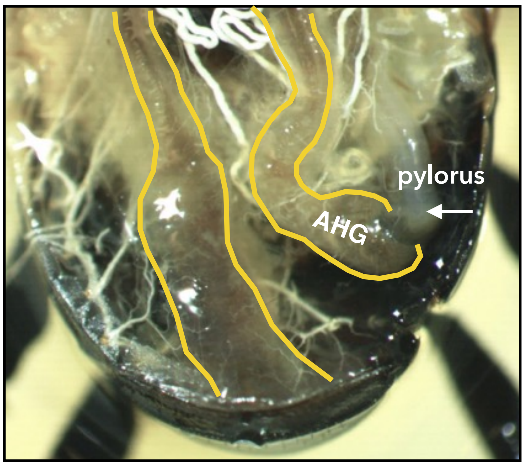

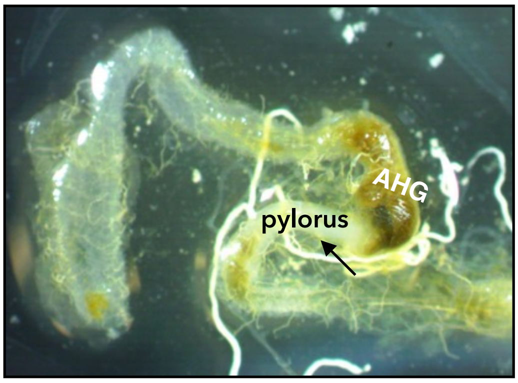

Fig.S8. *Mentophilus* hindgut.
Above: The *Mentophilus hollandieae* hind gut *in situ*. A small expanded region of the anterior hind-gut is visible just posterior to the ileum. Below: gut removed from abdominal cavity. A small expanded region is evident after the pylorus, but the hindgut is relatively short.

Table S9. Core bacterial families present in *Tesserodon*. Relative abundance of bacterial families is calculated from an average of two individuals of *Tesserodon pilicrepus*.

Phylum: CLASS: Family	average relative frequency	total for phylum
Bacteroidetes: BACTEROIDIA Porphyromonadaceae	15.6	39.9
Bacteroidetes: BACTEROIDIA Bacteroidaceae	13.7	
Bacteroidetes: BACTEROIDIA Rikenellaceae	10.6	
Firmicutes: NEGATIVICUTES: Acidaminococcaceae	2.0	21.1
Firmicutes: BACILLI: Enterococcaceae	0.2	
Firmicutes: CLOSTRIDIA Ruminococcaceae	16.6	
Firmicutes: CLOSTRIDIA Lachnospiraceae	1.4	
Firmicutes: CLOSTRIDIA Christensenellaceae	0.8	
Betaproteobacteria: Oxalobacteriaceae	0.4	0.4
Betaproteobacteria: Comamonadaceae	1.7	1.7
Proteobacteria: DELTAPROTEOBACTERIA: Desulfovibrionaceae	18.0	24.7
Proteobacteria: DELTAPROTEOBACTERIA: Rs-K70-termite group	6.7	
Planctomycetes: vadinHA49	8.8	8.8
Deferribacteres: DEFERRIBACTERES: Deferribacteraceae	0.8	0.8
Synergistetes: SYNERGISTIA: Synergistaceae	0.4	0.4
Archaea	1.6	1.6
total core	99.3	

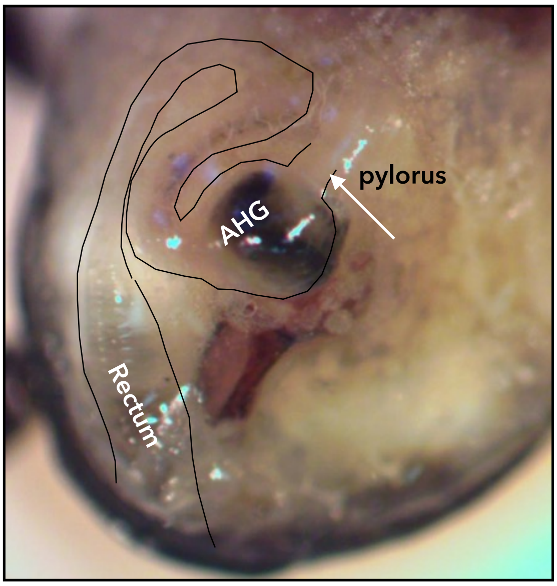

Fig.S9. *Tesserodon* hindgut. *Tesserodon pilicrepus* hindgut *in situ* shows a small expanded region of the anterior hind-gut, visible just posterior to the ileum.

Table S10. Core bacterial families present in dung-feeding *Onthophagus*. Relative abundance of bacterial families is calculated from an average of 92% of individuals (12 out of 13) from five species: *Onthophagus arrilla, O*. CQ2, *O. fuliginosus*, *O. granulatus*, *O. pugnax*.

Phylum: CLASS: Family	average relative frequency	total for phyum
Proteobacteria: BETAPROTEOBACTERIA: Comamonadaceae	1.8	1.8%
Proteobacteria: GAMMAPROTEOBACTERIA: Enterobacteriaceae	34.0	53%
Proteobacteria: GAMMAPROTEOBACTERIA: Pseudomonadaceae	19.0	
total % core	54.8	

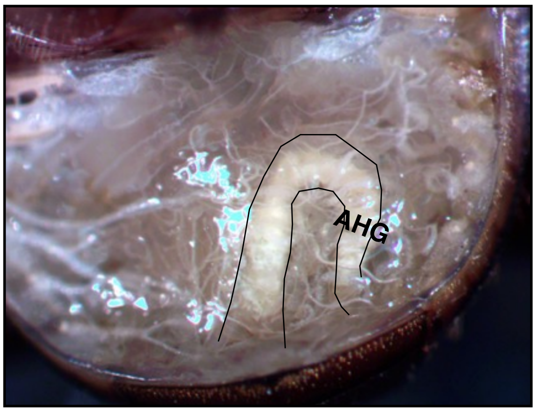

Fig.S10. *Onthophagus* hindgut. Hindgut of the dung-feeding *Onthophagus pugnax in situ*. The hind-gut is simple U-shape and short with a single bend; the white material was commonly seen in the *Onthophagus* gut.

Table S11. Core bacterial families present in mushroom-feeding *Onthophagus*. Relative abundance of bacterial families is calculated from an average of six individuals from two species: *O. dunningi* and *O. kumbaingeri*.

Phylum: CLASS: Family	average relative frequency	total for phyum
Bacteroidetes: CHITINOPHAGIA: Chitinophagaceae	0.5	7.1
Bacteroidetes: FLAVOBACTERIIA Flavobacteriaceae	6.0	
Bacteroidetes: BACTEROIDIA Porphyromonadaceae	0.6	
Proteobacteria: BETAPROTEOBACTERIA: Comamonadaceae	3.3	3.3
Proteobacteria: GAMMAPROTEOBACTERIA: Moraxellaceae	0.9	23.0
Proteobacteria: GAMMAPROTEOBACTERIA: Enterobacteriaceae	22.1	
Firmicutes: BACILLI: Erysipelotrichaceae	19.1	35.4
Firmicutes: BACILLI: Enterococcaceae	16.3	
total % core	68.8	

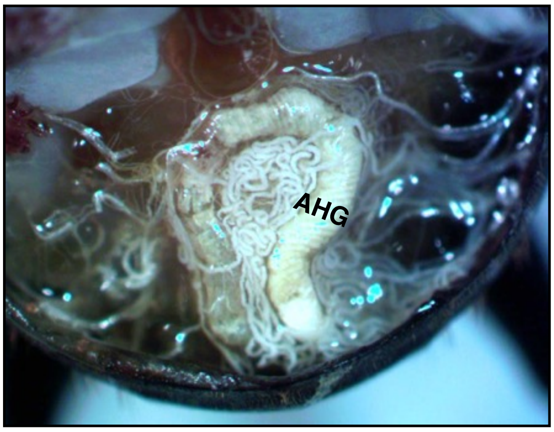

Fig.S11. *Onthophagus dunningi* hindgut. Hindgut of the mushroom-feeding *O. dunningi in situ*. The hindgut is a simple U-shape and short with a single bend; the white material was commonly seen in the *Onthophagus* gut.

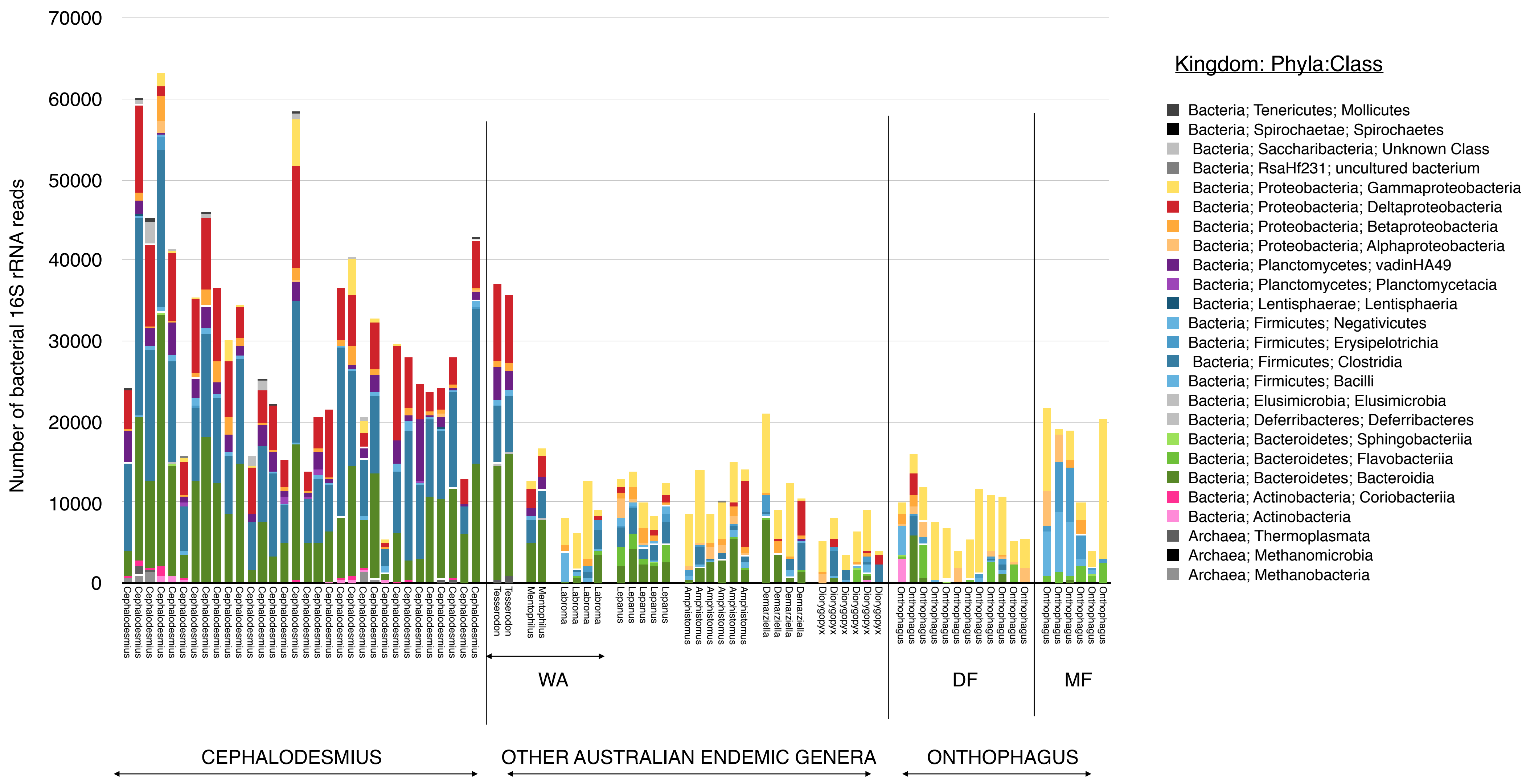

Figure S12. Number of bacterial 16S rRNA reads (Phylum:Class) in dung beetle gut samples. Members of the beetle genus *Cephalodesmius* are on the left. The other Australian endemic genera are separated into genera collected from Western Australia (WA) and Queensland. The *Onthophagus* gut community is shown on the right. DF indicates the dung-feeding Onthophagus and MF indicates the mushroom-feeding *Onthophagus*. The bacterial classes are colour-coded by phyla, for example, shades of green represent Bacteroidetes, yellow-orange-red are Proteobacteria, shades of blue represent Firmicutes purple represents Planctomycetes and brown and grey represent other minor classes.
